# What does scalar timing tell us about neural dynamics?

**DOI:** 10.3389/fnhum.2014.00438

**Published:** 2014-06-19

**Authors:** Harel Z. Shouval, Marshall G. Hussain Shuler, Animesh Agarwal, Jeffrey P. Gavornik

**Affiliations:** ^1^Deptartment of Neurobiology and Anatomy, University of Texas Medical School at HoustonHouston, TX, USA; ^2^Department of Neuroscience, Johns Hopkins UniversityBaltimore, MD, USA; ^3^Department of Biomedical Engineering, The University of Texas at AustinAustin, TX, USA; ^4^Department of Brain and Cognitive Sciences, The Picower Institute of Learning and Memory, Massachusetts Institute of TechnologyCambridge, MA, USA

**Keywords:** scalar timing, Weber's law, temporal intervals, temporal coding, neural dynamics

## Abstract

The “Scalar Timing Law,” which is a temporal domain generalization of the well known Weber Law, states that the errors estimating temporal intervals scale linearly with the durations of the intervals. Linear scaling has been studied extensively in human and animal models and holds over several orders of magnitude, though to date there is no agreed upon explanation for its physiological basis. Starting from the assumption that behavioral variability stems from neural variability, this work shows how to derive firing rate functions that are consistent with scalar timing. We show that firing rate functions with a *log-power* form, and a set of parameters that depend on spike count statistics, can account for scalar timing. Our derivation depends on a linear approximation, but we use simulations to validate the theory and show that *log-power* firing rate functions result in scalar timing over a large range of times and parameters. Simulation results match the predictions of our model, though our initial formulation results in a slight bias toward overestimation that can be corrected using a simple iterative approach to learn a decision threshold.

## 1. Introduction

Errors estimating the intensity of a stimulus commonly scale linearly with the magnitude of the stimulus. This relationship, called *Weber's Law*, has proven to be a surprisingly general property of the brain that accurately describes perception across sensory modalities (Weber, 1843; Coren et al., 1984). We have previously used basic principles to argue that this scaling naturally emerges if neural processes representing stimulus magnitudes have tuning curves with a specific mathematical form and that the generality of the law implies that this is a fundamental organizing principal of neural computation (Shouval et al., 2013).

An analog of Weber's law in the temporal domain, called *linear scaling* or *scalar timing*, states that errors estimating temporal intervals scale linearly with the duration of the intervals (Gibbon, 1977; Church, 2003). Temporal perception has been extensively studied by psychologists and neuroscientists for over 150 years, starting in the 1860s with Fechner (Fechner, 1966), leading to considerable knowledge about the behavioral aspects of temporal perception. Much less is known, however, about the underlying neural substrate responsible for engendering observed timing behavior.

Over the years many theories have been proposed to account for scalar timing. The scalar expectancy theory (Gibbon, 1977; Church, 2003) is based on a counter and accumulator model, conceptually similar to counting the ticks of a mechanical clock, and variability arises from comparison errors with remembered reference values. Another class of models assumes an ensemble of neurons oscillating at different frequencies, and timing is produced by decision neurons which become active only when a precise set of the oscillating neurons are coactive (Matell and Meck, 2000). These models are akin to a Fourier transform of the desired temporal response profile. Variability stems from the addition of stochastic noise to the ensemble dynamics, and it has recently been shown analytically that general addition of noise at various levels in the model can result in scalar timing (Oprisan and Buhusi, 2011, 2014). Note that these models are currently derived based on continuous dynamical systems, not spiking neural models. Drift diffusion models have also been proposed to provide a mechanistic basis of interval timing, though with spike-statistics that are inconsistent with scalar timing. Recent derivations of this model, with drift that is driven by opponent inhibitory and excitatory processes, can account for scalar timing (Rivest and Bengio, 2011; Simen et al., 2011). A final class of models, including this work, assume that timing is derived from the state of dynamic neural responses. For example, time can be estimated from the threshold crossing of decaying neural response (Staddon et al., 1999), or from a precisely designed set of leaky integrators (Shankar and Howard, 2012).

Here, by extending our earlier analysis (Shouval et al., 2013) to the temporal domain, we explore the relationship between neural dynamics and temporal perception and propose a theory of scalar timing based on experimentally verifiable physiological processes. Our approach is based on the assumption that estimates of a temporal interval vary on a trial-by-trial basis due to spike count variability (Dean, 1981; Tolhurst et al., 1983; Churchland et al., 2010). Although our analysis is mathematically homologous to the intensity variable case, the physiological substrates of time and intensity estimation are quite different. Here we show that neural processes with with activity levels that dynamically progress with a log-power temporal profiles can account for scalar timing, in much the same way that our previous work showed that Weber's law results from log-power tuning curves. Our analysis is based on a linear approximation, with results that are less precise than we found when analyzing intensity variables. Though the log-power model does produces scalar timing, our initial formulation of the model with the linear approximation results in a small bias toward underestimation and slightly less variability than found in simulations. In this paper we also derive a discrete approximation, which is applicable only in the temporal domain, that precisely estimates simulated neural variability. We also demonstrate that the bias is a consequence of our initial threshold selection criteria that can be easily eliminated with a simple algorithm that learns the correct threshold value to accurately decode desired intervals.

## 2. Methods and results

**Figure 1:**
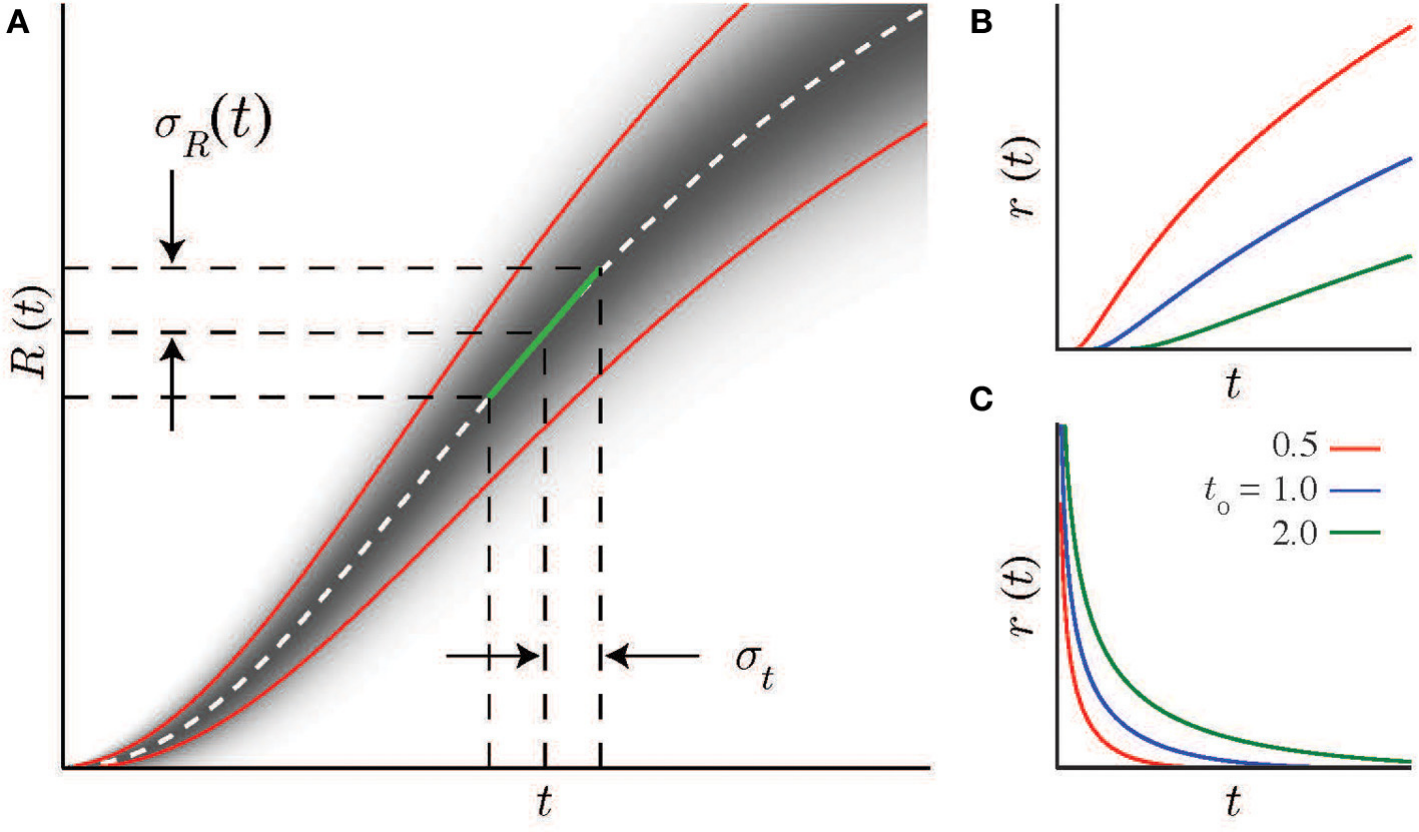
**Scalar timing and neural statistics**. **(A)** A local linear approximation (green line, Equation 2) of the the average firing rate *R*(*t*) (real distribution shown schematically by the gradient as a function of *t*, mean and standard deviations indicated by dashed-white and solid red lines) together with the scalar timing law leads to Equation 4, the solution of which (Equation 7 for the case of Poisson noise) is the firing rate curve *r*(*t*). Note, *R*′ is the slope of the linear approximation to *R*(*t*). **(B,C)** Example firing rate curves with Poisson spike statistics for different values of the integration constant *t*_0_. **(B)** Increasing solutions are defined above minimal values at *t*_0_. **(C)** Decreasing solutions are defined below maximal values at *t*_0_.

**Figure 2:**
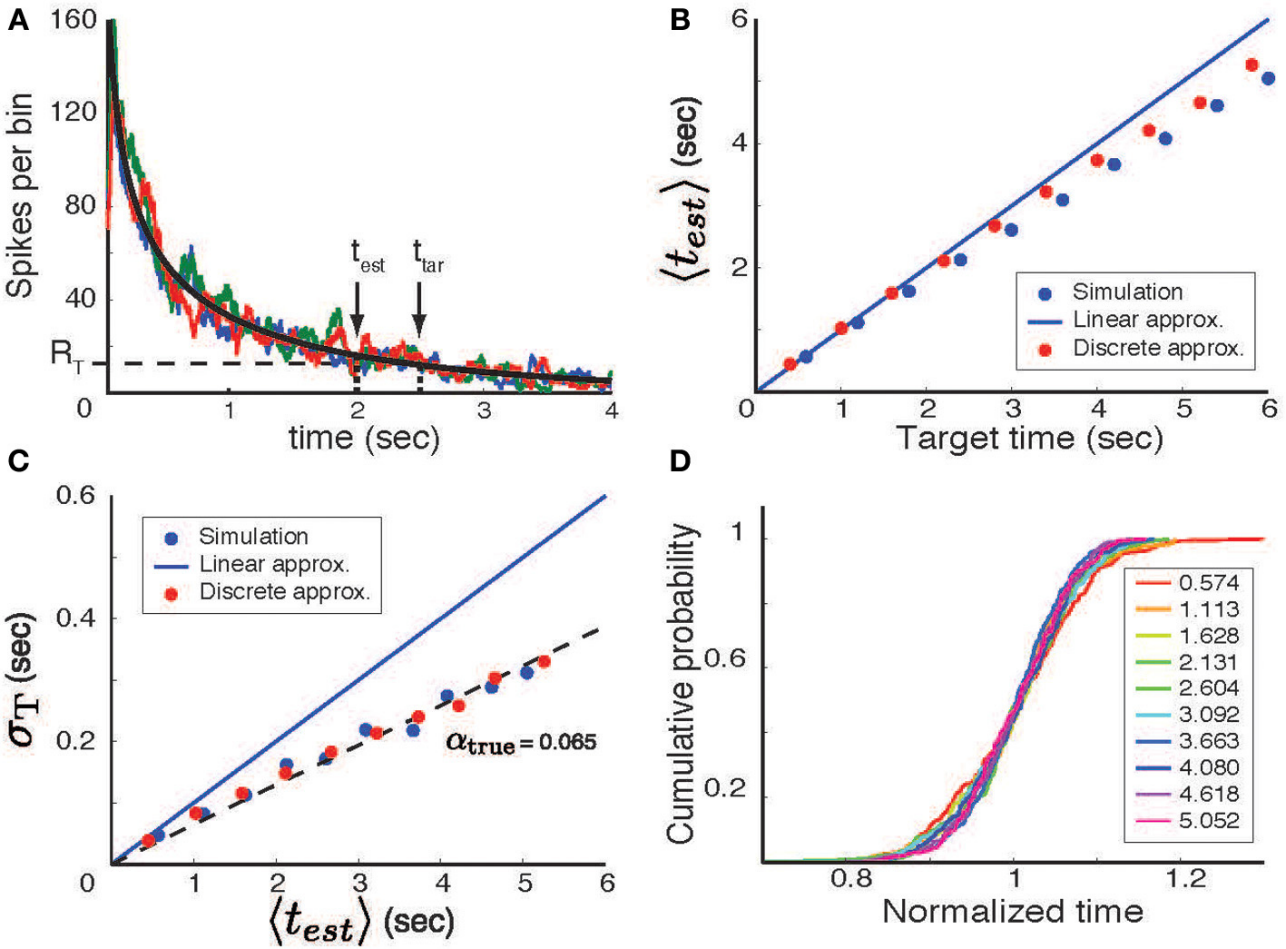
**Temporal interval estimation. (A)** A stimulus (at time *t* = 0) initiates a neural process with a mean firing rate (black line, determined by linear approximation theory) that decreases with time. In each trial the actual number of spikes varies stochastically; three trial-by-trial examples of the spike count variable are shown by the colored lines. The time estimate in each trial is determined by the first threshold crossing (*R*_*T*_ - horizontal dashed line) of the spike count variable. The actual estimated time for one trial (*t*_*est*_) is shown in comparison to the target time (*t*_*tar*_). **(B)** The mean time predicted by the model (〈*t*_*est*_ 〉, averaged over 200 trials) as a function of the target time. Blue circles based on simulations, red circles using discrete approximation. **(C)** The standard deviation of the time estimate (σ_*T*_) as a function of the mean predicted interval. **(D)** When rescaled by the mean estimated time (values specified by the color-code shown in the legend), the cumulative distributions of the actual response times overlap and are statistically indistinguishable (KS-test). These distributions were generated using Poisson statistics, a decreasing *log-power* function, *t*_0_ = 10 and τ = 0.1 sec.

We start with the assumption that the brain uses the temporal evolution of neural activity, which progresses with predictable stochastic dynamics, to estimate intervals. Specifically, we assume that some stimulus at time *t* = 0 initiates a neural process (describing either a single neuron or, more likely, a neural ensemble) that is characterized by a spike rate function *r*(*t*) (see Figures 1B,C), which either increases (Roitman and Shadlen, 2002) or decreases (Shuler and Bear, 2006) monotonically. The average spike count within a window τ is:

(1) $$R(t)=\int_{t-\tau /2}^{t+\tau /2}r(t+t^{\prime })dt^{\prime }$$

which can be approximated as *R*(*t*) ≈ τ*r*(*t*) for small τ values. As illustrated in Figure 2, the temporal interval described by this process is defined as the time required for *R*(*t*) to reach some threshold *R*_0_, which can be set as *R*_0_ = *r*(*t*_*tar*_) for a target time *t*_*tar*_. Noise driven fluctuations of *r*(*t*) result in variability of trial-by-trial estimates, *t*_*est*_, of the encoded target time.

In this framework there is a direct relationship between the magnitude of temporal estimate errors (which can be easily recorded using standard psychophysical methods) and spike count statistics that can be used to infer a mathematical form of *r*(*t*), and thus the underlying physiology, subject to the linear constraint on estimate errors as a function of *t*_*tar*_ specified by the scalar timing law. A simple linear approximation (illustrated in Figure 1A) of this relationship between the interval estimate and spike count has the form:

(2) $$\sigma_{t}(t_{est})\approx \frac{\sigma_{R}(t_{tar})}{\left|R^{\prime }(t_{tar})\right|}=\frac{\sigma_{R}(t_{tar})}{\left|\tau r^{\prime }(t_{tar})\right|}$$

where σ_*R*_(*t*) is the standard deviation of the average spike count at time *t*, and *R*′ (*t*_*tar*_) is derivative of the spike count curve with respect to the time, estimated at the target time. Note that σ_*t*_(*t*_*est*_) is the standard deviation of the estimation of the time *t* over many trials.

The scalar law states that errors estimating *t* scale linearly with *t*. Using standard deviation as the error measure:

(3) $$\sigma_{t}(t)=\alpha \;\cdot \;t.$$

where α specifies the slope of linear scaling, equivalent to the “Weber fraction.”

Combining Equations 2 and 3:

(4) $$\tau \frac{dr(t)}{dt}=\frac{dR(t)}{dt}=\pm \frac{\sigma_{R}(t)}{\alpha t}$$

where the + sign is valid when the slope of *r*(*t*) is positive and the − sign when it is negative.

We assume that spike count variability can be characterized using a power-law model with the form:

(5) $$\sigma_{R}(t)=\beta {\left(\tau r(t)\right)}^{\rho }$$

where the parameters β and ρ specify the specific noise model. This power-law model can account for many forms of spike-count variability. For example, ρ = 1/2 and β = 1 result in Poisson noise, and the ρ = 0 case is the constant noise case, which means spike count variability does not depend on the spike count. Experimentally spike count variability is found to be close to Poisson and often with somewhat larger variability than Poisson (ρ ≈ >1/2). Although the power-law noise is a relatively general model, it obviously cannot account for all forms of noise.

Applying this form to Equation 4, we obtain a differential equation relating the neural firing rate to specific noise and estimate error models:

(6) $$\frac{dr(t)}{dt}=\pm \left(\frac{\beta \tau^{\rho -1}}{\alpha }\right)\frac{r{\left(t\right)}^{\rho }}{t}$$

The solution of Equation 6 has a *log-power* form:

(7) $$r(t)=K\;\cdot \;{\left(\pm log(t/t_{0})\right)}^{n}$$

where $K=\frac{1}{\tau }{\left(\beta (1-\rho )/\alpha \right)}^{n}$, and $n=\frac{1}{1-\rho }$. This relationship holds whether *r*(*t*) rises (*t* ≥ *t*_0_, “+”case) or falls (*t* < *t*_0_,“−” case) monotonically. The integration constant *t*_0_ has a simple interpretation: it is the minimal (or maximal) time that can be estimated using this specific monotonically increasing (or decreasing) firing rate function (as shown in Figures 1B,C) for different values of *t*_0_. Note that all the parameters of the *log-power* function are determined by measurable spike statistics and behavioral performance; none of them are free parameters.

The specific shape of the general *log-power* form depends primarily on the spike count statistics. In the constant noise case (ρ = 0) this equation reduces to Fechner's law (Fechner, 1966). Hence, Fechner's law can be seen as making an implicit constant noise assumption. In the special and unrealistic case of proportional noise (ρ = 1) a power-law solution is obtained (Stevens, 1961).

Experimental recordings are often characterized by a nearly-linear relationship between mean spike count and variance(Dean, 1981; Tolhurst et al., 1983; Churchland et al., 2010). In the near-Poisson case, (ρ = 1/2), the *log-power* form has an exponent of *n* = 2 (note, the examples in Figures 1B,C assume Poisson statistics). We have previously shown for the case of magnitude estimation that Weber's law can be based on the tuning curves of either single neurons or neural ensembles (Shouval et al., 2013). Similarly here, while scalar timing can result if a single neuron's time-varying activity follows a *log-power* function, it is more likely to arise from the combined activity of a heterogenous population of neurons whose collective activity has the appropriate form.

To test the validity of our theory, we simulated a stochastic neural process with a monotonically falling spike rate in time (the increasing case, not discussed, is similar). Specifically, as per the derivations above, simulations were performed by generating spikes using a non-homogeneous Poisson process with a firing-rate parameter that decreased as a *log-power* function of time. Firing rates were determined by convolving the resultant spikes trains with a square window of width τ = 100 ms. The estimate of the temporal interval (*t*_*est*_) was defined, on a per-trial basis, as the time at which the firing rate first reached threshold (*R*_0_ set to *r*(*t*_*tar*_)). The result of these simulations are shown in Figures 2B,C. The mean value of *t*_*est*_ is close to, but a bit shorter than that predicted by the linear approximation theory (Figure 2B, blue circle) and the standard deviation is a linear function of the mean estimated time (Figure 2C–blue circle), although with a slope lower than that predicted by the linear approximation. Nevertheless, the rescaled distributions are almost completely overlapping (Figure 2D) and paired Kolmogorov-Smirnov tests find that the differences between these distributions are not statistically significant (although small differences may emerge with longer time spans, larger α values, or more trials). This shows that the *log-power* firing rate function indeed produces scalar timing, but that the theory described above results in an overestimate of error and a small bias of the mean.

It is possible to obtain a discrete approximation that better captures the simulation results. This approximation is obtained by dividing time into non-overlapping time bins of length τ, such the average spike count within a time bin (designated by the integer *i*) is *R*_*i*_ ≈ τ · *r*((*i* − 0.5) · τ). Under the assumptions that the bins are non-overlapping and have no significant temporal correlations, the spike counts in each bin are conditionally independent. Then, for a given spike generation model, the probability of a threshold crossing within a time bin as time unfolds is:

(8) $$P_{c}(i|R_{0})=\sum_{n\leq R_{0}}P_{s}(n|R_{i}),$$

where *P*_*s*_(*n*|*R*) is the probability of emitting *n* spikes given the mean spike count *R* (note that this formulation assumes a decreasing function *r*(*t*), for the increasing function the sum is over *n* ≥ *R*_0_). The probability that the first threshold crossing occurs in time bin *j* is

(9) $$P_{FC}(j|R_{0})=P_{c}(j|R_{0})\Pi_{i=1}^{j-1}(1-P_{c}(i|R_{0})).$$

This distribution can be used to calculate the mean, 〈*t*_*est*_〉, and standard deviation, σ_*T*_, of elapsed time estimates. Results of these calculations, (Figures 2B,C, red circles) agree closely with the numerical simulation results. The small discrepancy between this calculation and the actual simulations arises from partitioning time into non-overlapping time bins. Coarse grained simulations in which zero crossings are allowed only at these discrete points agree perfectly with the results of this discrete approximation.

**Figure 3:**
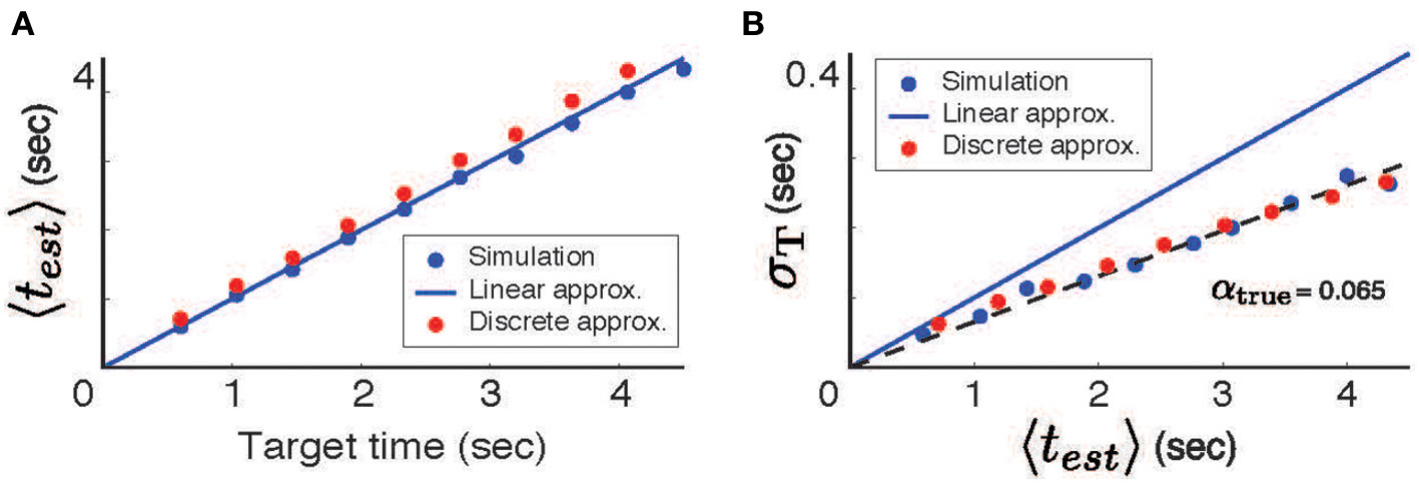
**Unbiased estimates obtained using a learned threshold**. **(A)** The mean estimated time (〈*t*_*est*_〉) as a function of the target time. **(B)** The standard deviation of the estimate error (σ_*T*_) as a function of the mean estimate.

Despite the close agreement between theory and simulations, the model as described consistently underestimates the mean target time. This bias, which results from the somewhat arbitrary decision to select *R*_0_ = *r*(*t*_*tar*_), can be corrected if the thresholds are learned rather than chosen directly from the spike count function. To learn the threshold (*R*_0_) we used a simple iterative learning rule:

(10) $$\frac{dR_{0}}{dt}=\pm \eta (t_{tar}=t_{est})$$

where the + sign corresponds to the monotonically falling firing rate case, and the − sign is used for the monotonically increasing cases, and η << 1 is the learning rate. This procedure quickly converges to provide an unbiased estimate of the target times (Figure 3A); error still scales linearly with time (Figure 3B) and the discrete approximation accounts well for the slope.

## 3. Discussion

Relating behavior to its underlying physiological mechanism is a fundamental aim of neuroscience. Here we have shown how, in a class of models based on the idea that time is estimated based on the dynamic state of neural processes, to relate scalar timing to the time varying firing rate of neurons. We show that, given firing rate statistics characterized by a power-law, scalar timing arises from a log-power firing rate function with parameters that depend on the spike statistics. Our derivation relies on a linear approximation, but we have also shown that a log-power function results in scalar timing irrespective of this approximation. The initial method for setting the detection threshold using the mean of the firing rate function causes a small estimate bias, but this can be corrected using an iterative procedure to find an appropriate detection threshold. Further, we have shown how to use a discrete approximation to calculate better estimates of encoded time and variability given a log-power firing rate function. These result depend on a mathematical analysis which is similar to the one used in our previous analysis of Weber's law for intensity variables (Shouval et al., 2013). Though it produces scalar timing, the linear approximation is less precise in the temporal domain than it was when we used it to analyze the coding of stimulus intensity. Accordingly, it was necessary to introduced a discrete approximation in order to accurately calculate simulated variability. We also showed how the decision-selection threshold can bias the encoded interval. Most models that account for Weber's law in the intensity domain are completely distinct from models that account for scalar timing. Our results show that a single mathematical approach can provide a unified explanation for these two distinct observations.

Although we derived firing-rate functions for the case of perfectly-linear scalar timing, the same procedure used to generate Equation 4 can also be used when if the relationship between estimation error and time is non-linear (Grondin, 2012). Similar derivations are possible for other functional forms of Equation 3. Indeed there are experiments showing that scalar-timing is precisely linear only in a limited range (Getty, 1975; Bizo et al., 2006; Grondin, 2012), and the exact forms of scaling observed in these experiments could be used to replace the linear scaling assumed here. There is no guarantee that an analytical result can be derived in such cases, but numerical solutions are always possible. Similarly, the same type of approach can be used to obtain a firing rate function, either analytically or numerically, assuming non-power-law forms for neural spike statistics.

Our analytical derivation produces a monotonically falling functions that are valid only below and upper threshold (*t*_0_) and monotonically increasing functions valid only above a lower threshold (*t*_0_). There is a lower threshold below which we can not evaluate time intervals, which would indicate that the monotonically increasing results are possibly more realistic. However, experimental results showing different Weber fractions at different temporal intervals could also be interpreted as indications that different processes are used for different time scales (Getty, 1975). One possibility is that falling functions could be used for very short temporal durations, on the order of a second or less, and increasing functions for longer durations.

As we outlined above, various models of interval timing have been proposed over the years to account for scaler timing (Gibbon, 1977; Matell and Meck, 2000; Church, 2003; Durstewitz, 2003; Oprisan and Buhusi, 2011, 2014; Rivest and Bengio, 2011; Simen et al., 2011; Shankar and Howard, 2012) and some share key properties of the model proposed here (Staddon et al., 1999; Durstewitz, 2003). An entirely different class of models is based on the idea that time can be read from the dynamic state of circuits in the cortical network (Buonomano and Mauk, 1994; Karmarkar and Buonomano, 2007), though the conditions for scalar timing in these models have not been analyzed. Some of the previously developed models of scalar timing are based on abstract entities such as counters and accumulators (Gibbon, 1977; Church, 2003), and some are dependent on continuous variables (Matell and Meck, 2000; Karmarkar and Buonomano, 2007; Oprisan and Buhusi, 2011), while others can be interpreted in terms of spiking inhibitory and excitatory neurons (Rivest and Bengio, 2011; Simen et al., 2011) and require nearly-perfect integration for the decision process. The model presented here is formulated directly in terms populations of spiking neurons with experimentally measurable variables. There are no free parameters in our model, since all depend directly on neural and behavioral statistics. Therefore, our theory has the advantage that it can be tested experimentally at the physiological level.

Our analysis indicates a very precise log-power form for the firing rate function. One might wonder, rightly, if it realistic to expect a neural processes to have such a precise formulation. It is important to realize that our analysis does not require or claim that any single neuron should display precise log-power dynamics, though to get true linear scaling the relevant population of neurons must possess this form. The population can be composed of individual neurons with diverse response dynamics, as we demonstrated in the intensity domain (Shouval et al., 2013). A question not answered here is how single neurons or a population of neurons can develop firing rate functions with a desired form. Possible answers are provided by previous work showing how single neurons with active conductances (Durstewitz, 2004; Shouval and Gavornik, 2011) or networks of interacting neurons (Gavornik et al., 2009; Gavornik and Shouval, 2011) can be tuned to, or even learn de-novo, specific temporal dynamics. An additional possibility is that decision neurons can select (in the Hebbian sense) a sub-population of existing neurons with a combined spike rate that has a log-power form without requiring that the dynamics of any of the individual neurons change at all, though we can not here propose a biologically realistic mechanism for making this choice.

Recent experiments (Leon and Shadlen, 2003; Shuler and Bear, 2006; Chubykin et al., 2013) have made physiological recordings from cortical cells in animals as they learn temporal discrimination tasks. These results show that the firing rate function of cells change when animals learn different temporal intervals and theoretical models have been devised to account for them (Reutimann et al., 2004; Gavornik et al., 2009). Analyzing these cases, and determining a single framework that leads to scalar timing, is quite different in many respects from the analysis carried out here. We are currently studying this issue. Experimentally it requires many trials to change the firing rate function. One possibility, as mentioned above, is that the model presented here describes mechanisms used to discriminate times in a manner that requires little or no learning, whereas other models would be required to describe how representations of specific temporal intervals are encoded over many trials. Regardless, the work here makes a strong prediction that any neural process used to encode temporal intervals that display scalar timing, with our minimal assumptions, will have firing rates that evolve as a log-power of time.

## Author contributions

Harel Z. Shouval and Jeffrey P. Gavornik developed the original work on scalar timing and Weber's law based on experimental work by Marshall G. Hussain Shuler. Harel Z. Shouval and Animesh Agarwal performed analysis and simulations, analysis was confirmed by Jeffrey P. Gavornik. Harel Z. Shouval and Jeffrey P. Gavornik wrote the paper with the help of and Marshall G. Hussain Shuler and Animesh Agarwal.

## Funding

This publication was partially supported by R01MH093665. Jeffrey P. Gavornik is supported by K99MH099654.

### Conflict of interest statement

The authors declare that the research was conducted in the absence of any commercial or financial relationships that could be construed as a potential conflict of interest.
